# Anti-inflammatory effects of ruxolitinib on chronic neutrophilic leukemia harboring *CSF3R-*T618I mutation with bilateral renal abscesses

**DOI:** 10.1016/j.lrr.2022.100348

**Published:** 2022-09-06

**Authors:** Naohi Sahara, Kazuaki Yokoyama, Takashi Matsunaga, Shinsuke Kitahara, Tomoki Fujii, Seiichiro Kobayashi, Nozomi Yusa, Eigo Shimizu, Seiya Imoto, Arinobu Tojo, Nobuhiro Ohno

**Affiliations:** a Department of Hematology, Kanto Rosai Hospital, 1-1 Kizukisumiyoshicho, Nakahara-ku, 211-8510 Kawasaki city, Kanagawa, Japan; b Department of Hematology/Oncology, Research Hospital, Institute of Medical Science, The University of Tokyo, 4-6-1 Shirokanedai, Minato-ku, 108-8639 Tokyo, Japan; c Department of Clinical Oncology, Kanto Rosai Hospital, 1-1 Kizukisumiyoshicho, Nakahara-ku, Kawasaki city, 211-8510 Kanagawa, Japan; d Department of Applied Genomics, Research Hospital, Institute of Medical Science, The University of Tokyo, 4-6-1 Shirokanedai, Minato-ku, 108-8639 Tokyo, Japan; e Health Intelligence Center, Institute of Medical Science, Research Hospital, Institute of Medical Science, The University of Tokyo, 4-6-1 Shirokanedai, Minato-ku, 108-8639 Tokyo, Japan

**Keywords:** Chronic neutrophilic leukemia, CSF3R-T618I mutation, Bilateral renal abscesses, Ruxolitinib

## Abstract

Chronic neutrophilic leukemia (CNL) is a rare myeloproliferative neoplasm (MPN) characterized by sustained mature neutrophilic leukocytosis. Recently, presence of colony-stimulating factor 3 receptor (*CSF3R*) mutations has been added to the diagnostic criteria for CNL. Anti-inflammatory effects of the JAK1/2 inhibitor ruxolitinib relieve constitutional symptoms associated with MPN, such as fatigue, night sweats, and fever. We present a case of CNL harboring *CSF3R-*T618I mutation exacerbated by concomitant bilateral renal abscesses, which was refractory to antibiotics, at the time of initial diagnosis. In this case, ruxolitinib rapidly improved not only CNL but the infection, due to its anti-inflammatory potency.

**Abbreviations**
aCML, atypical chronic myeloid leukemia
BM, bone marrow
CNL, chronic neutrophilic leukemia
CSF3R, colony-stimulating factor 3 receptor
JAK, Janus Kinase
MPN, myeloproliferative neoplasm
WHO, World Health Organization

## 1 Introduction

Chronic neutrophilic leukemia (CNL) is a rare myeloproliferative neoplasm (MPN) characterized by sustained mature neutrophils, hypercellular bone marrow (BM) without dysplasia, and few blasts. Though it can be difficult to distinguish, CNL diagnosis requires the exclusion of leukemoid reactions and other MPNs. The discovery of oncogenic mutations in the colony-stimulating factor 3 receptor (*CSF3R*) gene in most CNL patients has changed this [1]. The updated 2016 World Health Organization (WHO) classification includes *CSF3R* mutations as diagnostic criterion for CNL [2]. The most common mutation is T618I, a proximal membrane point mutation present in 88% of reported CNL cases [2]. Although there is no standard care or approved therapy for CNL, the Janus Kinase (JAK)1/2 inhibitor ruxolitinib induces good clinical responses in some patients harboring the *CSF3R-*T618I mutation [1,[3], [4], [5]]. In a large cohort of patients with myelofibrosis in phase-III studies, the anti-inflammatory effect of ruxolitinib resulted in pro-inflammatory cytokine reduction, leading to an improvement of constitutional symptoms, such as fatigue, night sweats, and fever [6,7]. Ruxolitinib also improved similar symptoms in some CNL patients in a phase-II study [4]. However, there have been very few reports of CNL with *CSF3R-*T618I mutation complicated by infections at the time of initial presentation and no reports of ruxolitinib being administered to such patients. Therefore, we present a case of *CSF3R-*T618I mutated-CNL exacerbated by concomitant bilateral renal abscesses that responded rapidly to ruxolitinib.

## 2 Case presentation

**Fig. 1:**
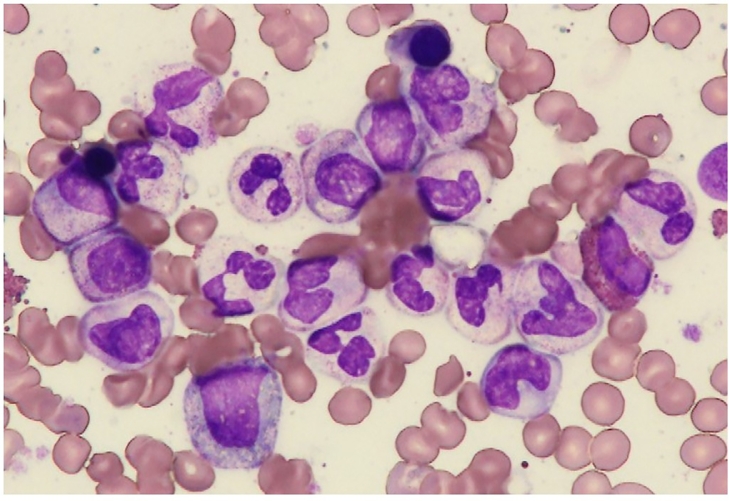
Bone marrow aspirate smear at diagnosis showing a hypercellular marrow with increased granulopoiesis. Neither dysplasia nor blast proliferation was observed. (Wright–Giemsa staining; original magnification, × 1000).

A 72-year-old man visited our hospital with a one-week history of persistent fever, fatigue, and lumbago in December 2019. His complete blood count showed a white blood cell count of 162 × 10^9^/L, hemoglobin of 8.5 g/dL, and platelet count of 220 × 10^9^/L. The differential count included 66% segmented cells, 15% stab cells, 5% metamyelocytes, 7% myelocytes, 4% promyelocytes, 1% blasts, and 2% monocytes. There was no evidence of dysgranulopoiesis. Serum lactate dehydrogenase and C-reactive protein (CRP) levels were elevated, 936 IU/L and 5.3 mg/dL, respectively. Physical examination revealed considerable splenomegaly, palpable 5 cm below the costal margin. BM examination revealed a marked hypercellular marrow with increased granulopoiesis without dysplastic features, and the blasts were 0.5% (Fig. 1).

**Fig. 2:**
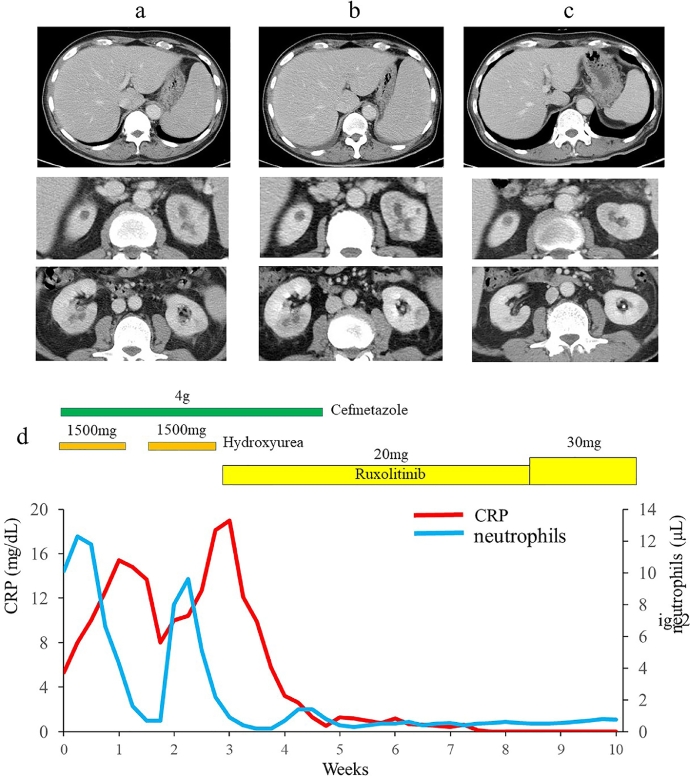
Contrast-enhanced computed tomography scans (a) on admission, (b) two weeks after hospitalization, and (c) three weeks after the initiation of ruxolitinib. (d) Clinical course of the present case.

Cytogenetic analysis revealed a normal karyotype. Fluorescence *in situ* hybridization for *BCR/ABL, PDGFRA, PDGFRB,* and *FGFR1* was negative. Mutation screening for *JAK2, CALR,* and *MPL* was also negative. To identify driver mutations, clinical sequencing was performed. This study protocol was approved by our institutional review board (No. 2020-1-0422) and was in accordance with the Declaration of Helsinki, and written informed consent was obtained from the patient. Whole-exome sequencing was performed and mutations in *CSF3R* (T618I), *ASXL1, TET2,* and *SRSF2* identified*.* Contrast-enhanced computed tomography (CT) revealed marked splenomegaly and multiple low-density areas in both kidneys (Fig. 2a).

Urinalysis showed many leukocytes, and urine culture detected methicillin-sensitive *Staphylococcus aureus* (MSSA), suggesting that the condition was complicated by bilateral renal abscesses. Since the number of neutrophil precursors, such as promyelocytes, myelocytes and metamyelocytes, in the peripheral blood, decreased to < 10% soon after antibiotics were initiated, these premature cells were considered to appear in response to the infection. CNL was diagnosed based on the revised 2016 WHO classification [2].

After admission, antibiotics (cefmetazole sodium) for MSSA and 1,500 mg/day of hydroxyurea to reduce neutrophils were started. Neutrophil levels decreased after one week, followed by decrease in fever and CRP levels; however, neutrophils levels surged and fever and CRP levels increased with withdrawal of hydroxyurea. CT revealed enlargement of the spleen and abscesses (Fig. 2b). Hydroxyurea was hence restarted, and once neutrophil levels normalized, it was replaced with ruxolitinib (20 mg/day). One day after starting ruxolitinib, the patient's fever and fatigue resolved remarkably, and CRP levels normalized two weeks later (Fig. 2d). Abdominal CT showed shrinkage of splenomegaly and multiple bilateral renal abscesses (Fig. 2c). After one month, ruxolitinib was increased to 30 mg/day and this dosage was continued. However, 10 months later, the patient developed blast transformation, despite repeated chemotherapy, and died in August 2021.

## 3 Discussion

CNL is an extremely rare MPN and has no dysplasia or any clinical or molecular criteria applicable to other MPNs [2]. Recent studies have demonstrated that somatic activating mutations in granulocyte *CSF3R* are detected in 80–100% of CNL cases [2]. Therefore, the latest revision of the WHO classification (2016) added the presence of *CSF3R* mutations to the major diagnostic criterion for CNL. The most frequently described mutation is the proximal membrane point mutation T618I representing 88% of *CSF3R*-mutated CNL cases [2]. Ligand-independent dimerization and *CSF3* activation leads to constitutive JAK/STAT signaling, which prompted the clinical use of the JAK1/2 inhibitor ruxolitinib [1]. There are several reports of patients with CNL with *CSF3R-*T618I mutation being treated with ruxolitinib [1,[3], [4], [5]]. In a phase II study of ruxolitinib in 21 patients with CNL and 23 with atypical chronic myeloid leukemia (aCML), the overall response rate of the *CSF3R*-mutated group was significantly higher than that of the wild-type *CSF3R* group [4].

This is the first case report of a patient with *CSF3R*-T618I-mutated CNL with antibiotic-resistant infection that demonstrated rapid response to ruxolitinib. To date, only one case of *CSF3R*-mutated CNL with concomitant infections has been reported; however, therapeutic strategies were not described in detail [8]. One of the reasons for the lack of infected cases is that for CNL diagnosis, leukemoid reactions and other MPNs (especially aCML) must be excluded; the fraction of circulating neutrophil precursors must be <10% [2]. However, when CNL is associated with severe infections, left-shifted premature granulocytes appear in the peripheral blood, making it difficult to accurately diagnose CNL. Therefore, identification of *CSF3R* mutations is essential for making an accurate diagnosis in infected cases of CNL, and to avoid missing out on ruxolitinib treatment.

Additionally, the ruxolitinib appeared to accelerate the improvement of severe infections in CNL. The JAK family of kinases plays a pivotal role in hematopoietic cell proliferation and differentiation, and is crucial for cytokine activation and signaling in the immune system. JAK2 is primarily correlated with hematopoietic growth factors, such as erythropoietin and thrombopoietin, and mediates differentiation and proliferation processes [9]. In contrast, the JAK1 isoform is mainly involved in the signaling of proinflammatory cytokines, such as interleukin (IL)-2, IL-6, and tumor necrosis factor alpha [9]. In MPN, while the anti-JAK2 inhibitory potency of ruxolitinib is responsible for controlling myloproliferation and reducing splenomegaly, its anti-JAK1 inhibitory property reduces pro-inflammatory cytokines, leading to improvement of constitutional symptoms, such as fatigue, night sweats, and fever [10]. The anti-inflammatory properties of ruxolitinib have changed the landscape in the treatment of graft versus host disease, the major complication of allogeneic stem cell transplantation [9]. And there are evidences supporting its clinical efficacy in improvement in MPN symptoms in patients with myelofibrosis [6,7] and CNL [4]. These reports support that in CNL exacerbated by antibiotic-resistant infection, ruxolitinib strongly suppressed inflammatory cytokines, resulting in rapid control of both CNL and infection. Since there are no reports on the use of ruxolitinib in cases of CNL complicated by infection, it remains unclear how ruxolitinib directly acted on bacterial infection in this case. Ruxolitinib may not only control neutrophil count but also improve neutrophil function. In particular, the renal abscess in this case, which is difficult for antibiotics to reach, might shrink due to ruxolitinib-mediated improvement in neutrophil migration ability. Sometimes the use of filgrastim can also produce similar "inflammatory" symptoms, especially in healthy donors, suggesting that CSFR stimulation can play a role in inflammation. Some reports indicate that neutrophil function increases in CNL [11], while others indicate that it decreases [12,13]. This discrepancy may be because these reports examined heterogeneous populations of CNL without *CSF3R-*incorporated diagnosis. To elucidate the role of ruxolitinib in CNL complicated by infection, it is necessary to study only CNL cases diagnosed using the revised WHO criterion.

In conclusion, ruxolitinib has the potential for early improvement of CNL exacerbated by infection due to its anti-cytokine ability. Therefore, it is important to promptly distinguish CNL from leukemic reactions and aCML by detecting *CSF3R*-T618I mutation, especially in cases of concomitant infections.

## Funding

This research did not receive any specific grant from funding agencies in the public, commercial, or not-for-profit sectors.

## Informed consent

Written informed consent to publish the case report was obtained from the patient's relative. This study was approved by the Ethics Committee of Kanto Rosai Hospital.

## Declarations of Competing Interest

The authors declare that there is no conflict of interest regarding the publication of this article.
